# The UK Standardisation of Breast Radiotherapy (START) Trial A of radiotherapy hypofractionation for treatment of early breast cancer: a randomised trial

**DOI:** 10.1016/S1470-2045(08)70077-9

**Published:** 2008-04-01

**Authors:** The START Trialists' Group

## Abstract

**Background:**

The international standard radiotherapy schedule for breast cancer treatment delivers a high total dose in 25 small daily doses (fractions). However, a lower total dose delivered in fewer, larger fractions (hypofractionation) is hypothesised to be at least as safe and effective as the standard treatment. We tested two dose levels of a 13-fraction schedule against the standard regimen with the aim of measuring the sensitivity of normal and malignant tissues to fraction size.

**Methods:**

Between 1998 and 2002, 2236 women with early breast cancer (pT1-3a pN0-1 M0) at 17 centres in the UK were randomly assigned after primary surgery to receive 50 Gy in 25 fractions of 2·0 Gy versus 41·6 Gy or 39 Gy in 13 fractions of 3·2 Gy or 3·0 Gy over 5 weeks. Women were eligible if they were aged over 18 years, did not have an immediate surgical reconstruction, and were available for follow-up. Randomisation method was computer generated and was not blinded. The protocol-specified principal endpoints were local-regional tumour relapse, defined as reappearance of cancer at irradiated sites, late normal tissue effects, and quality of life. Analysis was by intention to treat. This study is registered as an International Standard Randomised Controlled Trial, number ISRCTN59368779.

**Findings:**

749 women were assigned to the 50 Gy group, 750 to the 41·6 Gy group, and 737 to the 39 Gy group. After a median follow up of 5·1 years (IQR 4·4–6·0) the rate of local-regional tumour relapse at 5 years was 3·6% (95% CI 2·2–5·1) after 50 Gy, 3·5% (95% CI 2·1–4·3) after 41·6 Gy, and 5·2% (95% CI 3·5–6·9) after 39 Gy. The estimated absolute differences in 5-year local-regional relapse rates compared with 50 Gy were 0·2% (95% CI −1·3% to 2·6%) after 41·6 Gy and 0·9% (95% CI −0·8% to 3·7%) after 39 Gy. Photographic and patient self-assessments suggested lower rates of late adverse effects after 39 Gy than with 50 Gy, with an HR for late change in breast appearance (photographic) of 0·69 (95% CI 0·52–0·91, p=0·01). From a planned meta-analysis with the pilot trial, the adjusted estimates of α/β value for tumour control was 4·6 Gy (95% CI 1·1–8·1) and for late change in breast appearance (photographic) was 3·4 Gy (95% CI 2·3–4·5).

**Interpretation:**

The data are consistent with the hypothesis that breast cancer and the dose-limiting normal tissues respond similarly to change in radiotherapy fraction size. 41·6 Gy in 13 fractions was similar to the control regimen of 50 Gy in 25 fractions in terms of local-regional tumour control and late normal tissue effects, a result consistent with the result of START Trial B. A lower total dose in a smaller number of fractions could offer similar rates of tumour control and normal tissue damage as the international standard fractionation schedule of 50 Gy in 25 fractions.

## Introduction

In women with early breast cancer prescribed radiotherapy after tumour excision or mastectomy, the effective dose of radiation is adjusted to balance the risk of local cancer recurrence against the risk of harmful effects on healthy tissues. Radiotherapy reduces the risk of local relapse by about 70% and reduces breast cancer mortality.^1^ Such treatment is offered to nearly all patients after local tumour excision and to selected patients after mastectomy. The most frequently used schedule worldwide is 50 Gy, delivered in 25 fractions of 2·0 Gy over 5 weeks. This schedule has evolved pragmatically, and is based on an assumption that a high total dose delivered in small fractions of 2·0 Gy keeps the amount of normal tissue damage to a minimum while gaining the maximum level of tumour control. This perception was strengthened when early studies of hypofractionation, which did not use adequate reductions in total dose, reported unacceptably high rates of normal tissue injury.^2^

Normal and malignant tissues vary in their responses to radiotherapy fraction size, termed fractionation sensitivity. Responses are described by a model in which the sensitivity (measured by the degree of tissue damage for normal tissues, and tumour recurrence rates for malignant tumours) to fraction size is represented by the ratio of two constants α and β.^3^ The lower the ratio of α to β (expressed in Gy), the greater the effect on normal and malignant tissues of changes in fraction size. Healthy tissues of the breast and ribcage are sensitive to fraction size, with α/β values 5 Gy or less,^4^ so small changes in fraction size can produce relatively large changes in the effects of radiotherapy on these tissues. This sensitivity is typical of so-called late-reacting normal tissues that take months or years to develop atrophy or fibrosis after radiotherapy. By contrast, squamous carcinomas of the lung and the head and neck area have high α/β values (≥10 Gy), indicating low sensitivity to fraction size. In head and neck cancer, radiotherapy delivered in small fractions (≤2·0 Gy) to a high total dose spares late-responding normal tissues relative to tumour.^5^ Breast cancer has previously been thought to be insensitive to fraction size and best treated with fractions of 2·0 Gy or less. However, some trials have tested the hypothesis that breast cancer is as sensitive to fraction size as the normal tissues of the breast and underlying rib cage.^6^, ^7^, ^8^ If confirmed, these findings could indicate that small fraction sizes of 2·0 Gy or lower offer no therapeutic advantage, and that a more effective strategy would be to deliver fewer, larger fractions to a lower total dose.

Between 1986 and 1998 at the Royal Marsden Hospital (RMH) and Gloucestershire Oncology Centre (GOC) in the UK, 1410 patients prescribed whole breast radiotherapy after breast conservation surgery were randomised to 50 Gy in 25 fractions or to two dose levels of a 13-fraction regimen over 5 weeks—39 Gy in 3·0 Gy fractions and 42·9 Gy in 3·3 Gy fractions.^7^, ^8^ The primary endpoint was late normal tissue effects, with local tumour control as a secondary endpoint, and the results were consistent with breast cancer having a similar sensitivity to fraction size as the late-reacting healthy tissues. Based on this pilot study, the Standardisation of Breast Radiotherapy (START) Trials were initiated by the UK Coordinating Committee for Cancer Research (now National Cancer Research Institute) to extend the testing of radiotherapy schedules using fraction sizes larger than 2·0 Gy, in terms of local-regional tumour control, normal tissue effects, quality of life, and health economic consequences. The initiative consisted of two parallel trials, START Trial A and START Trial B. Trial A maintained the explanatory design of the RMH/GOC trial, with a reduction in one of the test group doses from 42·9 Gy in 3·3 Gy fractions to 41·6 Gy in 3·2 Gy fractions, following advice from the Independent Data Monitoring Committee. This change was recommended because the 42·9 Gy in 3·3 Gy regimen seemed to have slightly more late normal tissue effects than 50 Gy in 2·0 Gy fractions. The trial was designed to allow interpolation between two 13-fraction regimens in order to identify the schedule equivalent to 50 Gy in 25 fractions in terms of late normal tissue effects, and to compare local tumour control at this test-dose level. The protocol-specified intent was to combine the datasets of the pilot trial and START Trial A under guidance of the Independent Data Monitoring Committee to more precisely estimate the fractionation sensitivity of breast cancer. By contrast, START Trial B^9^ was a more pragmatic trial that compared a commonly used schedule in the UK (40 Gy in 15 fractions in 3 weeks) with a control group of 50 Gy in 25 fractions over 5 weeks. This paper presents the results of Trial A.

## Methods

Participation was open to all UK centres that provided radiotherapy treatment to patients with early breast cancer. With START Trials A and B running in parallel, centres chose to participate in either Trial A (17 centres) or B (23 centres). Due to earlier completion of recruitment in Trial B, those centres were invited to join Trial A after accrual to Trial B was complete.

### Patients

Women with operable invasive breast cancer (International Union Against Cancer pT1-3a pN0-1 M0) requiring radiotherapy after surgery (breast-conserving surgery or mastectomy, with clear tumour margins ≥1 mm) were eligible for the trial if they were aged over 18 years, did not have an immediate surgical reconstruction, and were available for follow-up. Unlike the pilot study, which had normal tissue effects as the primary endpoint, mastectomy patients were included in Trial A, since tumour control was one of the principal endpoints. Patients from 13 centres participating in Trial A were also recruited into the quality of life and health economics studies (health economics data not reported here, baseline quality of life data have been published elsewhere^10^). 13 centres recruited patients who had breast-conserving surgery into the photographic assessment substudy (12 centres were in both the quality of life and photographic studies). Patients from 12 centres also consented to donate a 20 mL blood sample for the study and to complete an associated family history questionnaire (substudy not reported here). The START Trials were approved by the South Thames Multi-Research Ethics Committee in September, 1998, and by the local ethics committees of all participating centres. Written informed consent was obtained for all patients.

### Procedures

START Trial A patients were randomised to either 50 Gy in 25 fractions (control group) or 41·6 Gy in 13 fractions or 39 Gy in 13 fractions (experimental schedules). All regimens were administered over 5 weeks to eliminate treatment time as a variable. This treatment involved five fractions per week in the control group and five treatments per fortnight (Monday, Wednesday, Friday one week, Tuesday and Thursday the next week) in each of the two experimental schedules.

Randomisation was arranged via telephone at the Clinical Trials and Statistics Unit at the Institute of Cancer Research (ICR-CTSU), Sutton, UK, where patient details were recorded and treatment was allocated. Randomisation was not blinded. Computer-generated random permuted blocks were used as the method of allocation, with patients stratified by hospital, type of surgery (breast conserving surgery or mastectomy), and intention to give a tumour bed boost dose or not. Use of adjuvant systemic treatment was recorded, with a requirement of at least a 2-week gap between exposure to chemotherapy and radiotherapy.

Patients lay in a supine treatment position. The planning target volume was defined as the whole breast with a 1 cm margin to palpable breast tissue; where regional radiotherapy was indicated, the planning target volume was supraclavicular nodes with or without axillary chain with a 1 cm margin. The decision to give regional radiotherapy was made before randomisation and was only used in 14% of patients (table 1). In two patients prescribed radiotherapy to the breast and supraclavicular fossa and randomised to the 41·6 Gy schedule, the total dose administered to the supraclavicular fossa was reduced to 39 Gy because of the sensitivity of brachial plexus to fraction size. Most patients were treated with 6 MV x-rays, although treatment with higher energies or cobalt γ-rays was allowed after discussion with the START Trial radiotherapy quality assurance team. Planning protocols were specified at the time of notification of participation into the study and had to conform to the minimum quality criteria described in the START Trial A protocol. Planning protocols varied slightly between centres, but within each centre they were identical in each fractionation group. Doses were prescribed to international reference points.^11^ Departments were required to have a protocol specifying whether patients who had breast-conserving surgery would receive a boost to the tumour bed, and to use an electron field of appropriate energy to deliver 10 Gy in five daily fractions to the 100% isodose after initial radiotherapy.

**Table 1:** Demographic and clinical characteristics at randomisation of the 2236 patients in START Trial A

		**Fractionation schedule**			**Total n=2236 (%)**
		50 Gy in 25 fractions n=749 (%)	41·6 Gy in 13 fractions n=750 (%)	39 Gy in 13 fractions n=737 (%)	
Age (years)					
	20–29	5 (0·7)	4 (0·5)	3 (0·4)	12 (0·5)
	30–39	38 (5·1)	40 (5·3)	38 (5·2)	116 (5·2)
	40–49	116 (15·5)	136 (18·1)	129 (17·5)	381 (17·0)
	50–59	280 (37·4)	283 (37·7)	286 (38·8)	849 (38·0)
	60–69	215 (28·7)	192 (25·6)	194 (26·3)	601 (26·9)
	70–79	87 (11·6)	85 (11·3)	78 (10·6)	250 (11·2)
	80−	8 (1·1)	10 (1·3)	9 (1·2)	27 (1·2)
	Mean (SD)	57·6 (10·5)	57·0 (10·7)	57·1 (10·5)	57·2 (10·6)
Time from surgery to randomisation (weeks); median (IQR) [range]		8·8 (5·3–20·8) [0·4–71·3]	9·4 (5·9–20·2) [1·0–50·3]	9·3 (5·4–21·1) [1·1–53·6]	9·1 (5·4–20·7) [0·4–71·3]
Primary surgery					
	Breast conserving surgery	631 (84·2)	641 (85·5)	628 (85·2)	1900 (85·0)
	Mastectomy	118 (15·8)	109 (14·5)	109 (14·8)	336 (15·0)
Histological type					
	Invasive ductal	581 (77·6)	585 (78·0)	584 (79·2)	1750 (78·3)
	Invasive lobular	88 (11·7)	95 (12·7)	83 (11·3)	266 (11·9)
	Mixed ductal/lobular	21 (2·8)	17 (2·3)	17 (2·3)	55 (2·5)
	Other	57 (7·6)	51 (6·8)	52 (7·1)	160 (7·2)
	Not known	2 (0·3)	2 (0·3)	1 (0·1)	5 (0·2)
Pathological node status					
	Positive	222 (29·6)	197 (26·3)	224 (30·4)	643 (28·8)
	Negative	514 (68·6)	536 (71·5)	497 (67·4)	1547 (69·2)
	Not known (no axillary surgery)	12 (1·6)	17 (2·3)	15 (2·0)	44 (2·0)
	Not known (missing data)	1 (0·1)	0 (0·0)	1 (0·2)	2 (0·1)
Tumour size (cm)					
	<1	24 (3·2)	26 (3·5)	24 (3·3)	74 (3·3)
	1−	362 (48·3)	347 (46·3)	355 (48·2)	1064 (47·6)
	2−	202 (27·0)	203 (27·1)	198 (26·9)	603 (27·0)
	3−	156 (20·8)	169 (22·5)	157 (21·3)	482 (21·6)
	Not known	5 (0·7)	5 (0·7)	3 (0·3)	13 (0·6)
Tumour grade					
	1	157 (21·0)	150 (20·0)	149 (20·2)	456 (20·4)
	2	369 (49·3)	379 (50·5)	368 (49·9)	1116 (49·9)
	3	212 (28·3)	207 (27·6)	210 (28·5)	629 (28·1)
	Not known (not applicable)*	11 (1·5)	10 (1·3)	6 (0·8)	27 (1·2)
	Not known	0 (0·0)	4 (0·6)	4 (0·5)	8 (0·4)
Adjuvant therapy					
	None	52 (6·9)	53 (7·1)	67 (9·1)	172 (7·7)
	Tamoxifen/no chemotherapy	416 (55·5)	418 (55·7)	376 (51·0)	1210 (54·1)
	Chemotherapy/no tamoxifen	86 (11·5)	77 (10·3)	82 (11·1)	245 (11·0)
	Tamoxifen+chemotherapy	173 (23·1)	187 (25·0)	188 (25·5)	548 (24·5)
	Other endocrine therapy†	17 (2·3)	13 (1·7)	17 (2·3)	47 (2·1)
	Not known	5 (0·7)	2 (0·2)	7 (0·9)	14 (0·6)
Lymphatic treatment					
	None	8 (1·1)	14 (1·9)	13 (1·8)	35 (1·6)
	Surgery/no radiotherapy	610 (81·4)	636 (84·8)	620 (84·1)	1866 (83·5)
	Radiotherapy/no surgery	3 (0·4)	4 (0·5)	2 (0·3)	9 (0·4)
	Surgery+radiotherapy	119 (15·9)	95 (12·7)	95 (12·9)	309 (13·8)
	Not known	9 (1·2)	1 (0·1)	7 (0·9)	17 (0·8)
Boost (BCS patients only)		n=631	n=641	n=628	n=1900
	Yes	381 (60·4)	391 (61·0)	380 (60·5)	1152 (60·6)
	No	242 (38·3)	249 (38·8)	241 (38·4)	732 (38·5)
	Not known	8 (1·3)	1 (0·2)	7 (1·1)	16 (0·8)
From baseline photographs		n=413	n=421	n=416	n=1250
Breast size					
	Small	43 (10·4)	47 (11·2)	41 (9·9)	131 (10·5)
	Medium	294 (71·2)	324 (77·0)	322 (77·4)	940 (75·2)
	Large	76 (18·4)	50 (11·9)	53 (12·7)	179 (14·3)
Surgical deficit					
	Small	232 (56·2)	235 (55·8)	249 (59·9)	716 (57·3)
	Medium	142 (34·4)	146 (34·7)	132 (31·7)	420 (33·6)
	Large	39 (9·4)	40 (9·5)	35 (8·4)	114 (9·1)

BCS=breast-conserving surgery.

* Lobular and other histological types.

† Other endocrine therapies include combinations of tamoxifen/anastrozole/letrozole/exemestane/goserelin, mostly within randomised trials.

All centres submitted details of the standard radiotherapy technique, after which a visit by the quality assurance team checked dosimetric measurements in a 2D and 3D breast phantom, including the junction region between supraclavicular fossa and tangential breast or chest wall fields.^12^, ^13^, ^14^, ^15^ The mean difference between prescribed and measured dose in a phantom was 2·1%. Additionally, a third of the radiotherapy treatment plans were collected and analysed by the quality assurance team to ensure compliance with the protocol in terms of prescription point, dose homogeneity, and lung depth. A random sample of patients had in-vivo thermoluminescent dosimeter measurements taken.^16^, ^17^, ^18^ The protocol allowed for a dose variation (in the planning target volume) between 95% and 105% of that at the reference point on the central axis. Lung depth data was obtained by the radiotherapy quality assurance programme, and analysis indicated that most patients had less than 2 cm of lung within the treatment volume. These results confirmed a good compliance with the technical aspects of the trial protocol.

The principal endpoints specified in the protocol were local-regional relapse, normal tissue effects, and quality of life. Local-regional tumour relapse was defined as local relapse in breast or chest wall, and regional relapse in ipsilateral axilla or supraclavicular fossa if it had been within an irradiated target volume. Any ipsilateral regional relapse outside the radiotherapy target volume was excluded from the analysis of local-regional relapse. Normal tissue effects in the breast, arm, and shoulder were assessed by photographic comparison with baseline, patient self-reported assessments, and physician assessments. Other endpoints were disease-free and overall survival, second primary cancers, and health economic consequences. Disease-free survival was defined as time to any breast cancer-related event (local-regional or distant relapse, contralateral breast cancer, or death from breast cancer). Data relating to five key breast normal tissue effects from the patient quality of life self-assessments are presented here. Separate papers will present the full analysis of all self-assessments and physician assessments of normal tissue effects, and of quality of life. Cases of ischaemic heart disease, symptomatic rib fracture, and symptomatic lung fibrosis were recorded during follow-up; incidence with and without confirmation of diagnosis (eg, using imaging and further investigation) was included. Brachial plexopathy was reported if damage to the brachial plexus was suspected and the patient had symptoms of pain, parasthesia, numbness, or other sensory symptoms (graded on a 4-point scale). Suspected cases of brachial plexopathy were subject to confirmation by neurophysiological assessment and MRI.

Patients were reviewed every year for tumour relapse and radiotherapy-induced normal tissue effects. Clinical data were recorded on pre-printed case report forms and sent to the coordinating clinical trials office at the ICR-CTSU, Sutton, UK. Photographs were taken at baseline (post-surgery and pre-radiotherapy) and then at 2 and 5 years to assess changes to the breast based on change in size, shrinkage, and shape, and scored on a 3-point graded scale. Changes in breast appearance (photographic) were scored by three observers blind to patient identity, treatment allocation, and year of follow-up, and a final agreed score reached by consensus. The assessment of change in breast appearance from photographs was fully validated in the pilot study.^7^ Breast size and surgical deficit were both defined from the baseline photographs by the same three observers applying 3-point graded scales. Quality of life data were obtained using standardised questionnaires^19^, ^20^, ^21^, ^22^ at baseline and at 6 months, 1, 2, and 5 years. Post-baseline quality of life questionnaires included an additional four protocol-specific items relating to changes in the affected breast after radiotherapy (skin changes in the area of the affected breast, overall change in breast appearance, firmness to touch of the affected breast, and reduction in size of the affected breast). Of these four items, patients who had had mastectomy only rated change in skin appearance after radiotherapy. Details of the quality of life study protocol and baseline data have been published elsewhere.^10^

The trial was coordinated by the ICR-CTSU, Sutton, UK. The trial was overseen by a Steering Committee of several independent experts joined by members of the ICR-CTSU, START Trial Management Group, and representatives of the funding bodies (as observers). The Trial Management Group was responsible for the day-to-day management of the trial, and the emerging safety and efficacy data was reviewed regularly by the Independent Data Monitoring Committee. Central statistical monitoring of data was done by ICR-CTSU, supplemented by selected on-site source document verification.

### Statistical analysis

The sample size was estimated with the intent of measuring differences in local-regional tumour relapse between each 13-fraction schedule and the control group. A 5-year local-regional tumour relapse rate of 10% in the 50 Gy group was predicted, based on the RMH/GOC pilot trial.^8^ A target sample size of 2000 patients was defined in Trial A to provide 80% power to detect a difference of 5% in the local-regional relapse rate between the control group and each test group (two-sided α=0·05). With this sample size, the estimated standard error for the difference between schedules was 1·64%.

Survival analysis methods were used to compare rates of each endpoint between the fractionation schedules. Length of follow-up was calculated as time from randomisation until time of first event or last follow-up assessment, whichever occurred first. Patients were still evaluable for local-regional relapse after distant relapse, but were censored at date of death. For the photographic endpoint, patients were no longer evaluable for change in breast appearance after local-regional relapse. Kaplan-Meier estimates of 5-year relapse rates, rates of normal tissue effects, rates of any breast-cancer related event, and mortality rates were calculated (with 95% CIs). For the patient quality of life self-assessments of normal tissue effects an event was defined as the first occurrence of a moderate or marked symptom (graded “quite a bit” or “very much”). The scores from the photographic assessments of change in breast appearance at 2 and 5 years were dichotomised as none versus mild or marked change, and the first occurrence of such a change was taken as the endpoint for the survival analysis. There were too few patients with marked change in breast appearance (photographic) to be able to analyse this category separately.

The Wald test was used to compare between pairs of fractionation schedules. Crude hazard ratios (with 95% CIs) comparing fractionation schedules for each endpoint were obtained from Cox proportional hazards regression models. The proportionality assumption of the Cox model was tested using Schoenfeld residuals and was found to be valid for all of the analyses. Since point estimates of differences in event rates can, by chance, be atypical of the overall pattern of differences between schedules, estimates of the absolute difference in 5-year event rates taking the whole range of observation times into account were obtained by applying the hazard ratios obtained from the Cox model to the Kaplan-Meier estimate of the rate in the 50 Gy control group.^23^ Both one-sided and two-sided 95% CIs were calculated for the absolute difference in local-regional relapse rates at 5 years, since the upper limit is of greater clinical interest, in view of concern about a possible excess risk caused by hypofractionated schedules. Kaplan-Meier survival curves and Nelson-Aalen cumulative hazard functions were plotted according to fractionation schedule. Plots were censored at the median length of follow-up (rounded to nearest year).

A direct estimate of the α/β value for breast cancer was obtained by fitting a Cox proportional hazards regression model containing terms for total dose, and total dose multiplied by dose per fraction, to the local-regional relapse data. Similarly, an estimate of the α/β value for dose-limiting normal tissues was obtained by fitting the same regression model to the photographic data for change in breast appearance. In each case, the α/β ratio was calculated by dividing the two parameter estimates (estimate for total dose/estimate for total dose × dose per fraction). Where appropriate, the Cox models included covariates associated with relapse and late normal tissue effects, so the resulting α/β value estimates (and 95% CI) were adjusted for prognostic factors. Approximate 95% CI for the α/β value estimates were calculated using a Taylor series expansion for the covariance of a ratio of two random variables. Lower limits of CIs for the α/β ratios were truncated at zero when the calculated limit was negative.

The protocol specified that the START Trial A data would be combined with the RMH/GOC pilot study data^8^ to obtain a combined estimate of the adjusted α/β values for breast cancer and for normal tissues. In view of the differences between the trials in terms of absolute rates of local relapse and patient characteristics, a meta-analysis was subsequently considered more appropriate, on the advice of the Independent Data Monitoring Committee. The meta-analysis was done by fitting Cox proportional hazards regression models using all individual patient data, and stratifying by trial. Known prognostic factors were included as covariates in the model. Repeating the meta-analysis by pooling the adjusted estimates from each trial (rather than using individual patient data) made little difference to the combined estimates (data not shown).

Analysis included all randomised patients on an intention-to-treat basis. This study is registered as an International Standard Randomised Controlled Trial, number ISRCTN59368779.

### Role of the funding source

The funding sources provided peer-reviewed approval for the trial and have had representation (as observers) on the Trial Steering Committee, but had no other role in the design, conduct, data collection, data analysis or interpretation of the study or the results. The corresponding author had full access to all the data in the study, and had final responsibility for the decision to submit for publication.

## Results

**Figure 1:**
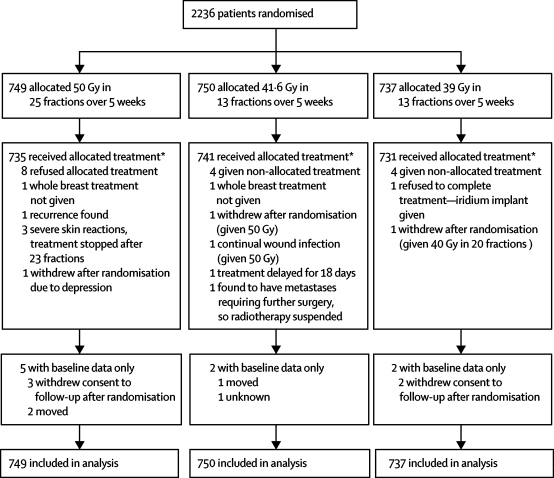
Trial profile for START Trial A *Only major treatment deviations listed. Minor deviations due to public holidays, machine service days, and machine breakdowns not included.

Between January, 1999, and December, 2002, 2236 patients were enrolled in START Trial A at 17 centres in the UK (figure 1). A total of 1129 patients enrolled in the quality of life study and 1306 in the photographic assessment study (with 878 patients enrolled in both substudies).

Demographic and clinical characteristics at randomisation were well balanced between treatment groups (table 1). Of the women prescribed chemotherapy, many (555/793, 70·0%) received an anthracycline-containing regimen, which was balanced between randomised radiotherapy schedules (178/259 [68·7%] for 50 Gy; 185/264 [70·1%] for 41·6 Gy; and 192/270 [71·1%] for 39 Gy). Cyclophosphamide, methotrexate, and fluorouracil combination therapy alone was prescribed in 227 women (28·6% of those receiving chemotherapy), which was similarly balanced between randomised groups (77 [29·7%] for 50 Gy; 78 [29·5%] for 41·6 Gy; and 72 [26·7%] for 39 Gy). 19 women (six for 50 Gy, nine for 41·6 Gy, and four for 39 Gy) received an adjuvant taxane. Of the 1805 women prescribed tamoxifen or another endocrine therapy, most (1790, 99·2%) were continuing treatment at randomisation (603/606 [99·5%] for 50 Gy; 611/618 [98·9%] for 41·6 Gy; and 576/581 [99·1%] for 39 Gy).

Data for oestrogen receptor status was not collected as part of START Trial A, but routine policy in most of the centres during the accrual period was to prescribe tamoxifen only to patients who were oestrogen-receptor positive or whose oestrogen receptor status was unknown. Hence tamoxifen use was a reasonable surrogate for oestrogen receptor status in the trial, which was balanced between the treatment groups. In the quality of life subgroup 21·6% patients underwent mastectomy. There were only 29 major treatment deviations (including early stopping of treatment, patient refusal of allocated treatment, and patients found to be ineligible for reasons including presence of relapses), resulting in 98·7% compliance with allocated treatment (figure 1). Compliance with completion of quality of life questionnaires over 5 years was more than 90%.

Median follow-up of surviving patients was 5·1 years (IQR 4·4–6·0), with a maximum follow-up of 8·0 years. At the time of analysis, 1881 patients (84·1%) were alive and without relapse, 36 (1·6%) were alive with local-regional relapse (without distant relapse), 54 (2·4%) were alive with distant relapse (including 14 with local-regional relapse), 256 (11·4%) had died (including 43 with local-regional relapse), and nine (0·4%) had no follow-up.

**Figure 2:**
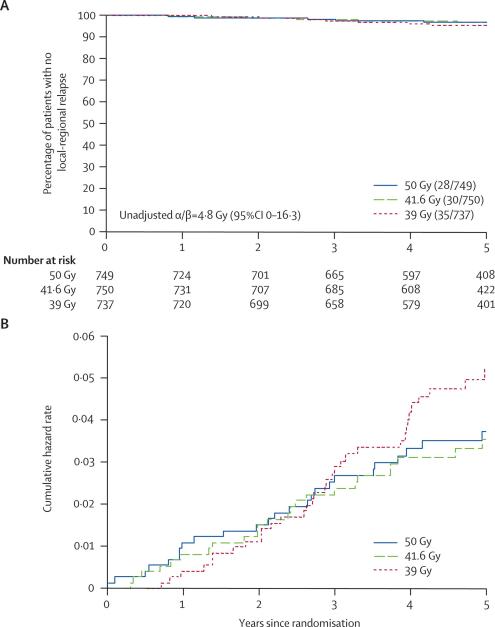
Kaplan-Meier plot (A) and Nelson-Aalen cumulative hazard plot (B) of local-regional tumour relapse in 2236 patients

At the time of analysis, 93 (4·2%) patients had had local-regional tumour relapse, and the hazard ratios relative to the 50 Gy group were 1·05 (95% CI 0·63–1·75) after 41·6 Gy and 1·26 (95% CI 0·77–2·08) after 39 Gy (table 2). The estimated absolute differences in local-regional relapse rates compared with 50 Gy at 5 years were 0·2% (95% CI −1·3% to 2·6%) after 41·6 Gy and 0·9% (95% CI −0·8% to 3·7%) after 39 Gy. Since the main concern over hypofractionation is an excess risk rather than a possible benefit, a more precise estimate of the potential excess risk of local-regional relapse was obtained from the upper limit of the one-sided 95% CI for the absolute difference in 5-year local-regional relapse rates for each 13-fraction schedule compared with 50 Gy. These indicated an estimated maximum 2·1% and 3·2% excess risk associated with 41·6 Gy and 39 Gy compared with 50 Gy, respectively. The Kaplan-Meier and cumulative hazard rate plots for local-regional relapse according to fractionation schedule (figure 2) illustrate the low event rate in all randomised groups.

**Table 2:** Survival analyses of relapse and mortality according to fractionation schedule in START Trial A

	**Events/total (%)**	**Estimated % with event by 5 years (95% CI)**	**Crude hazard ratio (95% CI)**	Wald test p value*
**Local relapse**†				
50 Gy	25/749 (3·3)	3·2 (1·9–4·6)	1	−
41·6 Gy	28/750 (3·7)	3·2 (1·9–4·5)	1·09 (0·64–1·88)	0·74
39 Gy	31/737 (4·2)	4·6 (3·0–6·2)	1·25 (0·74–2·12)	0·40
**Local-regional relapse**				
50 Gy	28/749 (3·7)	3·6 (2·2–5·1)	1	−
41·6 Gy	30/750 (4·0)	3·5 (2·1–4·3)	1·05 (0·63–1·75)	0·86
39 Gy	35/737 (4·7)	5·2 (3·5–6·9)	1·26 (0·77–2·08)	0·35
**Distant relapse**				
50 Gy	73/749 (9·7)	9·8 (7·5–12·0)	1	−
41·6 Gy	69/750 (9·2)	9·5 (7·3–11·7)	0·92 (0·66–1·28)	0·64
39 Gy	93/737 (12·6)	11·9 (9·5–14·4)	1·29 (0·95–1·76)	0·10
**Any breast cancer-related event**‡				
50 Gy	102/749 (13·6)	13·6 (11·0–16·2)	1	−
41·6 Gy	91/750 (12·1)	12·0 (9·6–14·5)	0·87 (0·65–1·15)	0·33
39 Gy	115/737 (15·6)	15·2 (12·5–17·9)	1·14 (0·87–1·49)	0·33
**All-cause mortality**				
50 Gy	84/749 (11·2)	11·1 (8·7–13·4)	1	−
41·6 Gy	89/750 (11·9)	11·3 (8·9–13·7)	1·04 (0·77–1·40)	0·81
39 Gy	83/737 (11·3)	10·7 (8·3–13·1)	1·00 (0·74–1·36)	0·99

* p value from Wald test comparing each schedule with 50 Gy.

† Local relapse defined as ipsilateral local tumour relapse in breast parenchyma/breast skin/chest wall skin.

‡ Breast cancer-related events: local, regional, or distant relapse, breast cancer death, contralateral breast cancer (disease-free survival).

The unadjusted α/β value for local-regional relapse estimated from a Cox proportional hazards regression model was 4·8 Gy (95% CI 0–16·3). Adjusting for prognostic factors resulted in extremely wide confidence limits, since the main effects for total dose and total dose × dose per fraction were not independently predictive of local-regional relapse in the regression model. Hence, the α/β value for local-regional relapse was left as a crude estimate.

Rates of distant relapse, disease-free survival, and overall survival were similar between the fractionation schedules, with no evidence of a clinically significant detriment for either of the hypofractionated schedules compared with 50 Gy (table 2).

**Figure 3:**
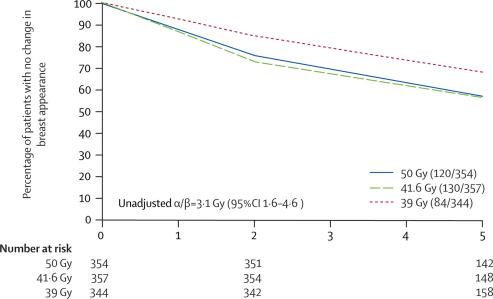
Kaplan-Meier plot of mild/marked change in breast appearance (photographic) in 1055 patients with breast conserving surgery

**Figure 4:**
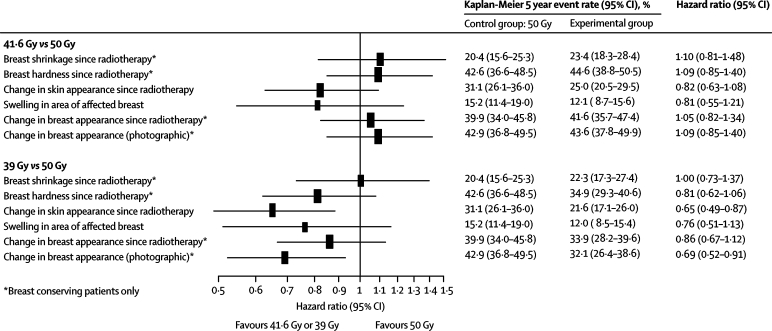
Forest plot of late normal tissue effects assessed as moderate/marked by patients and mild/marked from photographs

Change in breast appearance (photographic) was assessed in 1055 patients with both a baseline and at least one follow-up image (354 for 50 Gy, 357 for 41·6 Gy, and 344 for 39 Gy). Not all patients had photographs available at both 2 and 5 years, for reasons including the 5-year assessment not yet being due at the time of scoring and analysis, patient refusal, and withdrawal from the photographic study due to relapse. There were no associations between score for change in breast appearance (photographic) at 2 years or patient demographic or treatment characteristics and whether or not the patient had a 5-year assessment (data not shown). Mild changes were graded for 302 (28·6%) patients and marked changes for 32 (3·0%) patients, by 5 years. The hazard ratios for any (mild or marked) change in breast appearance compared with the 50 Gy group were 1·09 (95% CI 0·85–1·40, p=0·62) after 41·6 Gy and 0·69 (95% CI 0·52–0·91, p=0·01) after 39 Gy (Figure 3, Figure 4). Figure 3 shows that the treatment differences were evident at 2 years, and persisted to 5 years. The α/β estimate for any change in breast appearance (photographic) was 3·1 Gy (95% CI 1·6–4·6) adjusted for age, adjuvant therapy, lymphatic radiotherapy, breast size, and surgical deficit.

Patient quality of life self-assessments of late normal tissue effects were available for 1080 patients (95·7% of all patients in the quality of life study) with a baseline and at least one completed follow-up questionnaire (359 for 50 Gy, 364 for 41·6 Gy, and 357 for 39 Gy). Changes in breast appearance and breast hardness (breast conserving surgery patients) were the most common changes (figure 4). According to patient quality of life self-assessments of five key normal tissue effects on the breast or breast area, rates of moderate or marked effects by 5 years were similar after 41·6 Gy and 50 Gy. Rates of moderate or marked normal tissue effects tended to be lower after 39 Gy than after 50 Gy, with a significantly lower rate of change in skin appearance after 39 Gy than after 50 Gy (p=0·004). Figure 4 summarises the survival analyses of the photographic and patient quality of life self-assessments of late normal tissue effects for START Trial A, showing results generally in favour of the 39 Gy group compared with 50 Gy, and similar rates of effects after 41·6 Gy compared with 50 Gy.

The incidence of ischaemic heart disease, symptomatic rib fracture and symptomatic lung fibrosis was low at this stage during follow-up, and balanced between the schedules (table 3). In the 41·6 Gy group, there was one case of pneumonitis 9 months after treatment and one patient who developed mild symptoms and signs of brachial plexopathy 2 years after treatment. Two patients (both 50 Gy in 25 fractions) experienced an unusually marked acute skin reaction during their radiotherapy treatment, culminating in extensive moist desquamation. Neither of these patients had received adjuvant chemotherapy.

**Table 3:** Incidence of ischaemic heart disease, symptomatic rib fracture, and symptomatic lung fibrosis according to fractionation schedule

	**Fractionation schedule**			**Total n=2236 (%)**
	50 Gy n=749	41·6 Gy n=750	39 Gy n=737	
Ischaemic heart disease*				
Reported	12 (1·6)	7 (0·9)	8 (1·1)	27 (1·2)
Confirmed† [left-sided]‡	3 (0·4) [1]	2 (0·3) [0]	5 (0·7) [4]	10 (0·4) [5]
**Symptomatic rib fracture**§				
Reported	8 (1·1)	9 (1·2)	10 (1·4)	27 (1·2)
Confirmed†	1 (0·1)	2 (0·3)	1 (0·1)	4 (0·2)
**Symptomatic lung fibrosis**				
Reported	5 (0·7)	6 (0·8)	7 (0·9)	18 (0·8)
Confirmed†	0 (0)	2 (0·3)	1 (0·1)	3 (0·1)

Data are n (%).

* 18 patients had pre-existing heart disease at randomisation and were excluded.

† Cases confirmed after imaging and further investigations.

‡ Confirmed cases of ischaemic heart disease in patients with left-sided primary tumours.

§ Reported cases include three with rib fracture after bone metastases and nine after trauma.

There were 26 patients (1·2%) with contralateral breast cancer (13 after 50 Gy [1·7%], five after 41·6 Gy [0·7%], eight after 39 Gy [1·1%]), and 44 patients (2·0%) had other second primary cancers (15 after 50 Gy [0·7%], ten after 41·6 Gy [0·4%], 19 after 39 Gy [0·8%]), the most frequent being lung (six), ovarian (six), renal (four), and colorectal (four). The remaining 24 incidences of second primary cancers consisted of one or two cases of several different types.

When all patients in the RMH/GOC trial (1410 patients) and START Trial A (2236) were included in a meta-analysis, the unadjusted estimate of the α/β value for local-regional relapse was 4·1 Gy (95% CI 0·9–7·4). Adjusting for known prognostic factors (age, chemotherapy, tamoxifen, lymphatic radiotherapy, type of primary surgery, boost, and pathological tumour size) gave an adjusted α/β value for local-regional relapse of 4·6 Gy (95% CI 1·1–8·1). Including all the 1202 RMH/GOC trial and 1055 START Trial A patients with available photographic assessment data in a meta-analysis, the unadjusted estimate of the α/β value for any change in breast appearance was 3·6 Gy (95% CI 2·4–4·9). Adjusting for age, chemotherapy, tamoxifen, breast size, and surgical deficit gave an adjusted α/β value of 3·4 Gy (95% CI 2·3–4·5).

## Discussion

The results of START Trial A are consistent with the hypothesis that breast cancer is as sensitive to fraction size as the normal tissues. The first indication that breast cancer could be safely and effectively treated using fraction sizes above 2·0 Gy was first raised more than 20 years ago, when biological models were applied to retrospective clinical data.^24^, ^25^ In START Trial A, 41·6 Gy in 13 fractions was similar to the control regimen of 50 Gy in 25 fractions in terms of normal tissue effects and also in terms of local tumour control.^24^, ^25^ This result is consistent with that of the START Trial B, in which 40 Gy in 15 fractions over 3 weeks seemed at least as safe and effective as 50 Gy in 25 fractions. The combined trials present mounting evidence that hypofractionation is a safe and effective approach to breast cancer radiotherapy.

The fractionation sensitivity of breast cancer is quantified by an α/β ratio of 4·8 Gy, but the small number of local-regional tumour relapse events (N=93) limits precision (95% CI 0–16·3 Gy). Statistical power is enhanced by considering the results of START Trial A together with a larger number of local-regional relapses (N=185) recorded by the pilot trial over a 15-year period.^8^ A meta-analysis of the pilot data and START Trial A generates an α/β ratio for breast cancer of 4·6 Gy (95% CI 1·1–8·1). The confidence limits of such an estimate are still fairly wide but closer to the estimate of α/β ratio for normal tissue effects of 3·4 Gy (95% CI 2·3–4·5) from the meta-analysis of the photographic assessments. Despite the residual imprecision in estimating fraction size sensitivity, breast cancer seems to respond differently to human squamous carcinomas of the bronchus and head and neck region, and to experimental animal tumours characterised by α/β values of 10 Gy or more.^5^ This distinction is important, not only because it has potential implications for breast cancer treatment, but also because it contributes to accumulating evidence that cancers vary in their sensitivity to fraction size.^26^ The molecular mechanisms governing fractionation sensitivity are not yet understood, but the cellular correlates include recovery from sub-lethal and potentially lethal damage.^27^ The ability to predict fractionation sensitivity from the tumour phenotype or genotype might allow fraction size to be adjusted more accurately to the individual cancer.

The initial estimate of a 10% rate of local-regional relapse at 5 years in the 50 Gy control group was based on the pilot trial started in 1986. Since then, improvements in surgery, radiotherapy techniques, systemic therapy, and more effective multidisciplinary working have reduced risks of both local and metastatic breast cancer relapse. The fall in local relapse rates over the years is excellent news for patients. While accrual was still continuing, the emerging data presented in confidence to the Independent Data Monitoring Committee suggested that relapse rates were likely to be lower than predicted. At that time, adhering to appropriate governance procedures, the potential effects of the predicted lower than expected relapse rates were discussed by the independent Trial Steering Committee and the Independent Data Monitoring Committee. The consensus from these advisory committees was that there was no strong scientific rationale for changing the protocols and increasing the sample size. Since the local-regional relapse rate was lower than expected (<4%), the study actually has greater power than originally planned to detect a 5% absolute difference (97% power, assuming 4% in the control group), which represents a much larger relative treatment effect (4% in control group *vs* 9% in test group compared with 10% *vs* 15% as specified in the protocol). Alternatively, with the lower baseline local-regional relapse rate the same sample size provides sufficient power to detect smaller absolute differences. For example, with a 4% local-regional relapse rate in the control group, the study is able to detect an absolute difference of 3·5% with a similar level (82%) of power as originally planned.

The photographic assessments of normal tissue effects confirm a dose-response relation between the two test doses, from which a precise estimate of α/β value has been derived, consistent with previous published work.^27^ Assuming a conservative α/β value of 3 Gy for all normal tissue effects except nerve injury, the 39 Gy and 41·6 Gy test doses are equivalent to 46·8 Gy and 51·6 Gy in 2·0 Gy equivalents, respectively. The fractionation sensitivity of underlying lung tissue or heart might differ from that of ribcage and breast, but this uncertainty is less relevant now that the heart can usually be physically shielded when advanced techniques of radiotherapy planning and delivery are applied.^28^ The results of patient quality of life self-assessments of normal tissue effects in START Trial A are largely consistent with the dose response effect seen in the photographic assessments.

Physician assessments of normal tissue effects have not been presented in this paper. Preliminary analysis of these data produce estimates of the relative effects of the fractionation schedules which seem similar to those assessed by the photographic and patient self-assessments. Some variation existed, however, between centres in the practice used to complete the yearly case report forms. Most centres completed these forms in the presence of the patient, whereas others completed them afterwards from hospital case notes. Since the level of detail included in the hospital case notes varied within and between centres, this practice, although unbiased between treatment groups, could have led to underreporting of physician-assessed normal tissue effects. The results of the physician assessments will thus be the subject of a separate paper, reporting also the sensitivity of the endpoints according to method of completion of case report forms.

The 11·5% absolute difference in breast appearance at 5 years between the two test regimens generates a γ value of 1·4 at the steepest part of the dose-response curve (the γ value measures the gradient of the dose response curve as the percent increase in effect per percent increase in total dose delivered in 2·0 Gy fractions^29^), which converts into a 5·2% absolute increase in normal tissue effects per 2·0 Gy fraction. The local-regional relapse data from START Trial A generate a γ value of 0·2, which gives some indication of the small gains expected from dose escalation when tumour control is more than 95%.^30^ This finding has implications for modelling the imprecision in the estimate of breast cancer fractionation sensitivity. Taking into account the higher 10-year local-regional relapse rates in the RMH/GOC pilot trial, the current precision of the α/β estimate can be shown by estimating a 95% CI for the 10-year local-regional relapse rate of a specific fractionation schedule. If the α/β value for tumour control is as high as 8·1 Gy (the upper 95% confidence limit), a 13-fraction regimen matched to 50 Gy in 25 fractions for late normal tissue effects would have an anti-tumour effect equivalent to 47·3 Gy in 2·0 Gy fractions, accounting for 1·5% more local-regional relapses compared with 50 Gy in 25 fractions. If the α/β value for tumour control is as low as 1·1 Gy (the lower 95% confidence limit), the same schedule would be responsible for 4·3% fewer local-regional relapses at 10 years compared with 50 Gy in 25 fractions. With the lower 5-year local-regional relapse rates in START (3–4%) compared with the RMH/GOC pilot trial (8%), the absolute excess local-regional is roughly half in the START patient population.

A median follow-up of 5 years is too short to allow assessment of all the potential late normal tissue effects such as cardiac damage. Follow-up of all women within the trial is continuing in order to assess the long-term effects of the fractionation schedules. However, the RMH/GOC pilot data (median follow-up 10 years) showed that the relative effects of different fractionation schedules remain unchanged over time. 15–20 years of follow-up will be needed to reliably measure cardiac effects. The short-term priority is to protect the heart from exposure to radiotherapy; something that is now possible with advanced radiotherapy technologies.

In conclusion, our data are consistent with the hypothesis that breast cancer shows similar responsiveness to fraction size as the late responding normal tissues of the breast, as indicated by the α/β estimates. A 13-fraction regimen is unlikely to represent the limits of hypofractionation. The ongoing NCRN FAST Trial is testing five fractions of 5·7 Gy and 6·0 Gy, treating one fraction per week, with a long-term aim to reduce overall treatment time, not just for convenience to patients, but to minimise the potential effect of rapid tumour growth during radiotherapy.
